# Performance Evaluation of Fast Microfluidic Thermal Lysis of Bacteria for Diagnostic Sample Preparation ^†^

**DOI:** 10.3390/diagnostics3010105

**Published:** 2013-01-17

**Authors:** Michelle M. Packard, Elizabeth K. Wheeler, Evangelyn C. Alocilja, Maxim Shusteff

**Affiliations:** 1 Nano-Biosensors Laboratory, Michigan State University, 115 Farrall Hall, East Lansing, MI 48824, USA; E-Mails: packard2@msu.edu (M.M.P.); alocilja@msu.edu (E.C.A.); 2 Lawrence Livermore National Laboratory, 7000 East Ave, Livermore, CA 94450 USA; E-Mail: wheeler16@llnl.gov

**Keywords:** thermal lysis, microfluidics, sample preparation, on-chip diagnostics, lab-on-a-chip, bacterial detection

## Abstract

Development of new diagnostic platforms that incorporate lab-on-a-chip technologies for portable assays is driving the need for rapid, simple, low cost methods to prepare samples for downstream processing or detection. An important component of the sample preparation process is cell lysis. In this work, a simple microfluidic thermal lysis device is used to quickly release intracellular nucleic acids and proteins without the need for additional reagents or beads used in traditional chemical or mechanical methods (e.g., chaotropic salts or bead beating). On-chip lysis is demonstrated in a multi-turn serpentine microchannel with external temperature control via an attached resistive heater. Lysis was confirmed for *Escherichia coli* by fluorescent viability assay, release of ATP measured with bioluminescent assay, release of DNA measured by fluorometry and qPCR, as well as bacterial culture. Results comparable to standard lysis techniques were achievable at temperatures greater than 65 °C and heating durations between 1 and 60 s.

## 1. Introduction

Significant efforts are being expended on making diagnostic technologies usable and relevant in point-of-need and point-of-care (POC) settings, such as low-resource environments [1,2]. Microfluidic technologies have a number of advantages in this context, including small sample and device size, low power and fast assay times [3]. As nucleic-acid based assays proliferate and mature, a means for cell lysis typically needs to be incorporated to release DNA and/or RNA from the cell prior to analysis [4,5]. Traditional benchtop cell disruption by agitation with silica beads (“bead-beating”) is highly effective, but entails the typical drawbacks of batch-processing and manual sample handling required with microcentrifuge-tube-based methods. A range of approaches have been attempted to miniaturize and adapt lysis methods for downstream microfluidic molecular detection of bacteria, including ultrasonic [6,7], physical disruption [8,9], photothermal [10], optolysis [11], electrolysis [12,13,14,15,16,17,18] and chemical lysis [13,19,20,21,22]. Most of these methods require the addition of beads, nanoparticles or chemical regents. Addition of these reagents may impede detection by interfering with detectable molecules or inhibiting assay processes (e.g., PCR), so additional purification steps are required. For instance, one ultra-high-temperature thermal-only approach [23] used a 95% ethylene glycol buffer, which required subsequent dilution to avoid inhibiting downstream PCR. Thus, further development of methods that do not impose additional purification requirements is advantageous. Thermal lysis is a highly robust and simple technique that does not require any additional sample preparation and can readily be integrated with other microfluidic processes.

The major advantage of the device used in this work is that it allows heat-only lysis in a single-step flow-through manner, without introducing reagents or particles. Though some cell types, such as bacterial endospores or human spermatozoa, are well-known to be heat resistant, detailed investigation into the results of heat lysis with heat-susceptible cell types are worthwhile due to the simplicity of this method. Here, we take a first step in this direction by processing pure cultures of the Gram-negative enterobacterium *Escherichia coli* (*E. coli)* over a range of temperatures and flow rates. This work can lead to a more detailed understanding of processes taking place at the molecular level during bacterial lysis and permeabilization, by analyzing lysates using a number of different assays. The results of lysis were assessed by measurement of membrane compromise, protein release, DNA release and culturability. Each assay yields slightly different information about the state of the bacterial cells after heat treatment. The aggregated picture that emerges provides insight into the physical processes taking place due to the elevated temperature. 

## 2. Experimental Section

### 2.1. Bacterial Sample Preparation

*E. coli* C3000 (ATCC Cat. No. 15597) were incubated overnight in LB broth, then centrifuged at 4,500 ×*g* for five minutes at room temperature, rinsed in deionized water and re-suspended in TE buffer (Teknova, 10 mM Tris, 1mM EDTA, pH 8.0) prior to chip delivery. The approximate starting concentration of 10^7^ cfu/mL was determined by plate culture, as described in Section 2.5.

### 2.2. Microfluidic Chips

**Figure 1:**
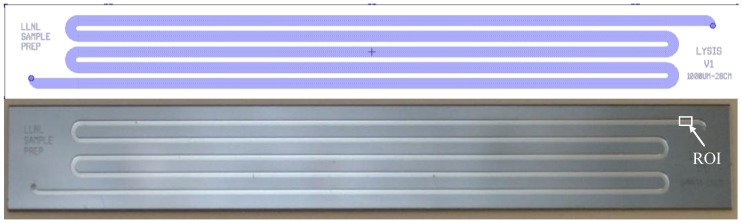
Silicon and glass microfluidic lysis chip—layout in Tanner L-edit software (**top**) and finished device (**bottom**). The top device has a 1 mm wide channel and the bottom device an 0.5 mm wide channel. The external chip dimensions are 70.5 mm × 9 mm × 1 mm. The rectangle marked “ROI” denotes the imaging area used for the BacLight live:dead membrane permeabilization assay (see Section 2.6).

Cells were lysed on-chip via a multi-turn serpentine microchannel (Figure 1) fabricated using standard photolithography and bulk micromachining methods. Fluid channels with a simple rectangular cross-section were etched into <100> silicon wafers using deep reactive ion etching (DRIE). Fluid access ports were through-etched from the back side of the wafer, also by DRIE, and the channels were sealed by anodic bonding (350 °C, constant voltage −900 V, ~5 min) to borosilicate glass. The channel dimensions were designed to provide on-chip volumes between 17 and 84 µL, with maximal residence times up to 50 s at ≈100 µL/min flow. The particular device used to generate the data reported here had channel dimensions of 0.5 mm × 0.3 mm × 17 cm, resulting in an internal volume of 25 µL. The serpentine design was chosen to ensure substantial axial fluid velocity, as compared to a wide single-pass chamber design, which both reduces non-specific adsorption of material on the interior walls, as well mitigates potential temperature non-uniformities.

The chips were heated by a Kapton^®^ KHLV series (Polyimide Film and PSA) rectangular resistive heater (KHLV-0502/10, 28 V, 1 × 5 cm, 1.6 W/cm^2^) clamped to the silicon side of the chip, and a type K thermocouple positioned between the chip and heater provided feedback to a temperature controller (Alpha Omega Instruments Series 800). An estimate of the temperature gradient through the thickness dimension of the device was made based on heat diffusion calculations, indicating that the temperature on the opposite side of the chip is within <2 °C and within the fluid layer itself, within 1 °C, which is acceptable precision for these experiments. Additionally, the high thermal conductivity of silicon (≥150 W/m·K) allows the reasonable assumption of temperature uniformity within 1 °C in the bulk of the silicon chip in the lateral directions. 

### 2.3. Experimental Procedure

**Figure 2:**
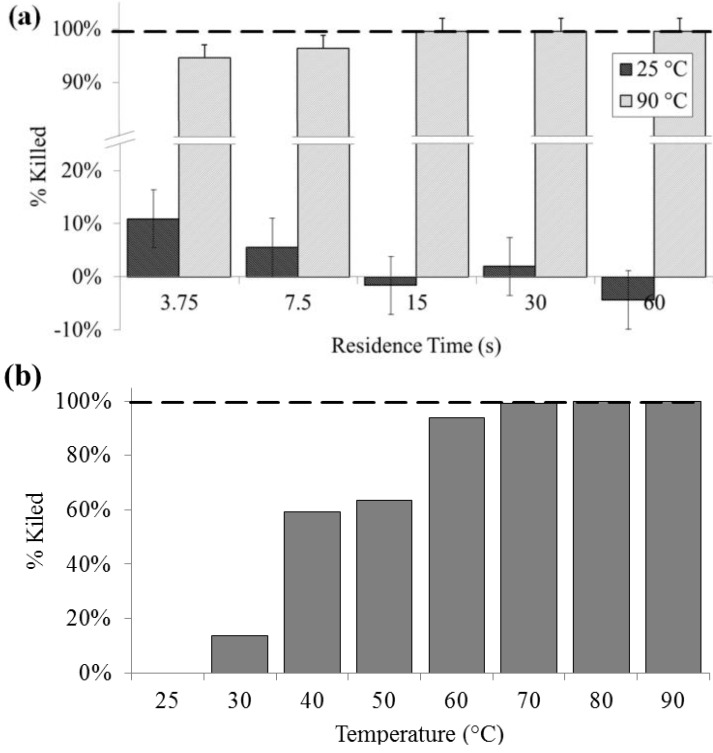
Lysis performance as indicated by a decrease in colony forming units compared with a bead-beaten positive control (– – –). (**a**) For a range of on-chip residence times from 15 to 60 s at room temperature (25 °C) and at maximum heat (90 °C). (**b**) For the full temperature range for 15 s residence time each.

**Figure 3:**
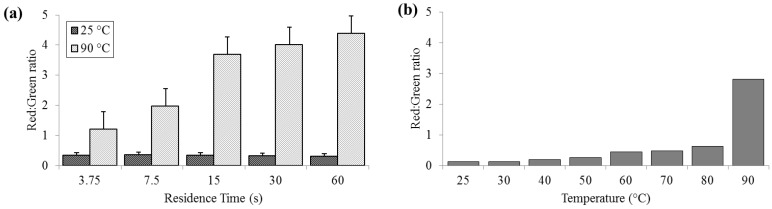
Membrane permeability as detected by the Baclight live/dead assay and measured as red:green ratios. (**a**) For a range of on-chip residence times from 15 to 60 s at room temperature (25 °C) and maximum heat (90 °C). (**b**) For the full temperature range for 15 s residence time each.

**Figure 4:**
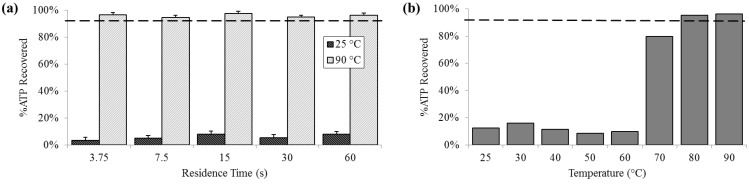
Percent extracellular ATP determined by chemiluminescent signal compared with a bead-beaten positive control (– – –). (**a**) For a range of on-chip residence times from 15 to 60 s at room temperature (25 °C) and maximum heat (90 °C). (**b**) For the full temperature range for 15 s residence time each.

**Figure 5:**
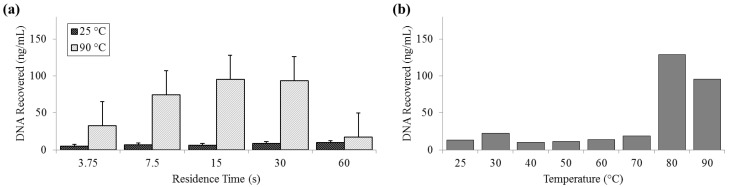
Quantity of DNA recovered as reported by Qubit PicoGreen assay. (**a**) For a range of on-chip residence times from 15 to 60 s at room temperature (25 °C) and maximum heat (90 °C). (**b**) For the full temperature range for 15 s residence time each.

**Figure 6:**
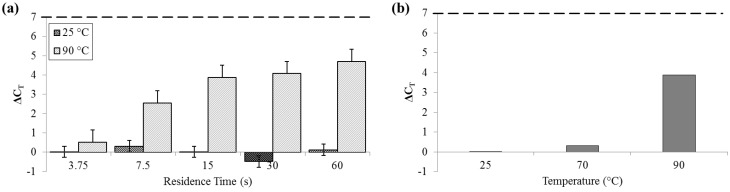
ΔC_T_ by qPCR compared to bead-beaten control (– – –). (**a**) For a range of on-chip residence times from 15 to 60 s at room temperature (25 °C) and maximum heat (90 °C). (**b**) For three temperatures, each with a 15 s residence time.

Lysis experiments were carried out by first entering a temperature setpoint on the controller and allowing the water-filled chip to equilibrate. Samples were then pumped through the chip using a syringe pump (Cole Parmer) at the flow rate required to give a specified on-chip residence time (400, 200, 100, 50 and 25 µL/min for the time-series of Figure 2, Figure 3, Figure 4, Figure 5 and Figure 6). The syringe pump connection to the chip inlet was a short length of PFA tubing (≈10 µL), with a second length of tubing (≈15 µL) connected to the outlet carrying the processed sample directly to a collection vial for post-lysis analysis. The first 150 µL of the effluent for each experiment were discarded, and a volume of 500 µL was subsequently collected. Calculations of the timescale for heat diffusion into and out of the sample fluid revealed that the sample requires <100 ms to reach the temperature of the surrounding walls, while passing through the lysis chip and the outlet tubing. This implies that the temperature equilibration time when entering and exiting the chip is negligible relative to the on-chip residence time. For the data reported here, the heating duration is therefore assumed to be equal to the on-chip residence time. 

### 2.4. Bead Beating (Positive) Controls and Negative Controls

For each set of measurements, a positive control experiment was carried out by “bead-beating” the bacteria—physically disrupting the sample by agitation with 100 µm glass beads (BioSpec Mini-BeadBeater, 4800 RPM, 180 s, approx. 1 mL each sample and beads). This sample was used as a reference, representing the maximum expected signal to which results from thermal lysis are compared. 

In addition to the bead-beaten positive controls, negative control measurements were also made to ensure that lysis signals did not arise due to nonspecific adsorption in the microfluidic chip and tubing. For the Invitrogen Baclight and Promega BacTiter assays, background fluorescence and luminescence signals were subtracted for baseline correction. For culture, Qubit and PCR measurements, negative control experiments showed blank samples giving signals below the detection threshold.

### 2.5. Bacterial Culture

To assess effects of on-chip lysis on bacterial culturability, each collected sample was serially diluted in distilled water (dilution range between 10^3^ and 10^5^) then immediately plated on Tryptic Soy Agar and incubated overnight. Plates were then imaged and counted manually to compare with culture counts from unprocessed samples. 

### 2.6. Membrane Permeabilization

For an assessment of bacterial membrane integrity, permeabilization was monitored *in situ* by staining with Baclight live/dead assay (Invitrogen) consisting of SYTO^®^-9 and propidium iodide (PI). Both dyes fluoresce only when bound to the minor groove of cellular DNA. SYTO^®^-9 is a green, membrane-permeant dye, capable of entering both live and dead cells, whereas PI enters cells only upon membrane disruption. The dyes were added to the bacterial samples prior to chip delivery to allow real-time quantification on a Zeiss Axiovert 5100 fluorescent microscope equipped with a 485/20 nm excitation filter and 505 nm long-pass emission filter. Images were acquired near the chip outlet, after the sample had passed through the heated region (region marked “ROI” in Figure 1, approximate image area 730 × 550 µm), using a ScopeTek DCM200 2.0M pixel CCD camera and MiniSee software, with 100 ms exposures. Fluorescent signal was then analyzed by summing over the entire image area in the red and green color channels using ImageJ software [24] to quantify the ratio of red:green signal as a function of residence time and temperature. Quantitative analysis was carried out in Microsoft Excel.

### 2.7. Protein Release

Protein release was measured by detection of extracellular ATP via the Promega BacTiter-Glo^TM^ Microbial Cell Viability Assay (Cat. No. TB337). Samples were spun at 4,500 ×*g* for ten minutes, after which the supernatant was separated from the pellet, and the pellet was resuspended in distilled water. Relative luminescent units for the supernatant (RLU_SUP_) and resuspended pellet (RLU_PEL_) were measured on a single-tube luminometer (Berthold). Percent extracellular ATP was then calculated as a fraction of the total (RLU_SUP_)/(RLU_PEL_ + RLU_SUP_).

### 2.8. Release of DNA

DNA release was quantified in two ways: by fluorometry using the Qubit system (Invitrogen) with the dsDNA intercalating dye PicoGreen (Invitrogen) and by real time quantitative PCR (qPCR). Specific qPCR for *E. coli* was performed on a BioRad iCycler using TaqMan primers and FITC-labeled probes, using a protocol with an initial 4-min hot-start at 95 °C, followed by 40 cycles of denaturation for 15 s at 95 °C, annealing for 30 s at 60 °C and extension for 30 s 72 °C. The primer and probe set was designed with binding sites in a transporter gene region conserved in *E. coli* strains and showed no cross-reactions with any other organisms, including genetic near neighbors, *Salmonella enterica* and *Shigella flexneri*. To ensure that only released DNA is being quantified, samples were spin-filtered prior to measurement through low-binding microcentrifuge tube filters (Pall Nanosep, OD100C34, 100000 MWCO) to remove unlysed cells and debris. Positive control bead-beaten samples were similarly spin-filtered. Therefore, no additional cell lysis is expected due to the initial hot-start step in the PCR protocol.

In a qPCR reaction, C_T_ is the number of doubling cycles required to reach a threshold signal level and, thus, a measure of the DNA content at the start of the reaction. The most informative metric for the qPCR data in this work is the difference in threshold cycle (ΔC_T_) comparing lysed to unlysed samples. The C_T_ of the unprocessed sample is used as the baseline, while ΔC_T_ values for samples processed through the lysis chip at various temperatures and flow rates are reported relative to this baseline. 

## 3. Results

For the results presented below, each data point represents a single measurement. In panel (a) of each figure, except where specified otherwise, error bars were calculated as follows to estimate experimental variation and measurement uncertainty: for the data series at 25 °C, the size of the bars is the standard deviation of 25 °C data values at all flow rates (N = 5); for the data series at 90 °C, the bars are the standard deviation of three measurements performed at on-chip time of 15 s and 90 °C (N = 3). In panel (b) of each figure, no error bars are shown, but a realistic upper bound on the uncertainty is the error bar of the corresponding 90 °C data series. Error bars are shown single-ended for clarity, and all uncertainties are assumed to be ± symmetric. Further investigation will be necessary to increase statistical robustness. 

### 3.1. Culture

Culturability was significantly decreased at lysis temperatures of 40 °C and above, and a 100% loss of colony forming units was noted above 60 °C. Lysis at 90 °C as determined by colony count was above 90% (SD = 0.2%) for all residence times. For all flow rates at 25 °C, there was no significant effect on culturability, except at 400 µL/min, indicating that bacteria are generally not lysing due to shear, rather than heat. Lysis due to flow-induced shear may be another rapid on-chip lysis approach that bears further investigation. In contrast, complete loss of culturability was achieved on chip at 15 s residence time or greater (Figure 2).

### 3.2. Cell Membrane Permeability

Significant cell membrane permeabilization, as indicated by increased ratios of red to green signal, (summed over the entire image area) was measured at 90 °C for residence times as short as 3.75 s; however, sixty seconds were required to achieve maximal change. Red:green ratios (R_R/G_) were reliably less than 0.5 for bacteria held at 25 °C (SD_25_ = 0.02, N = 3) and increasing to levels near 5.0 (SD_90_ = 0.58) at 90 °C (Figure 3). According to the manufacturer’s protocol for the live/dead assay, for 100% live *E. coli*, the minimum expected R_R/G_ is ≈0.312, while the maximum R_R/G_ for 100% dead *E. coli* is between 9 and 10 (with R_R/G_ = 4.5 corresponding to approximately 95% dead). These values are approximate, since the red and green channels in a color image do not strictly correspond to the spectral regions specified by Invitrogen. 

### 3.3. Protein Release

At 25 °C, the percent of ATP detected extracellularly was less than 10% (SD_25_ = 2.0%), compared with almost 80% at 70 °C and increasing to above 95% (SD_90_ = 1.4%) 80 °C and 90 °C. Extracellular ATP was reliably above 95% at 90 °C, regardless of residence time (Figure 4).

### 3.4. Release of DNA

DNA release was measured by both fluorometry (Qubit) and qPCR. Qubit measurements of DNA recovered at 25 °C remained constantly below 10 ng (SD_25_ = 2.01 ng) and peaked at almost 130 ng (SD_90_ = 32.6 ng) at 80 °C. The fluorometer measurement of the PicoGreen signal decreased at 90 °C and was notably low at long residence times, suggesting that there is a decrease in detection efficiency by PicoGreen post-denaturation (Figure 5).

For measurements of DNA release by quantitative polymerase chain reaction (qPCR), ΔC_T_ was not significant for samples that flowed through the device at 25 °C (SD_25_ = 0.29 cycles). Comparatively, at 90 °C, ΔC_T_ was greater than 4 cycles (SD_90_ = 0.62 cycles), indicative of an approximately 20× increase in PCR-available DNA (Figure 6).

**Figure 7:**
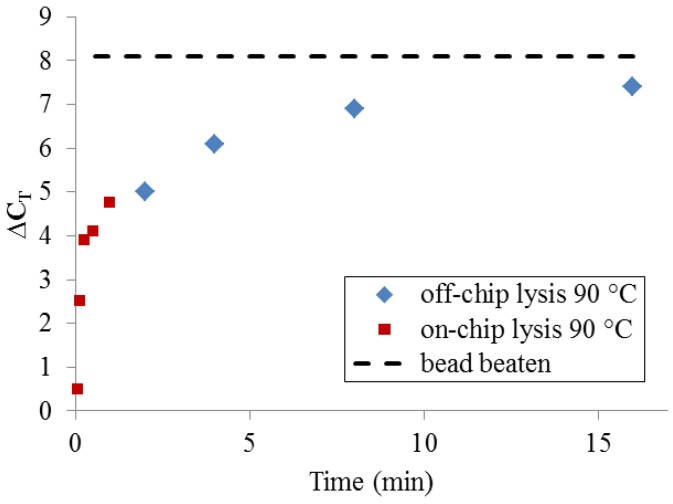
PCR-available DNA yield relative to an unprocessed sample (ΔC_T_ = 0), resulting from long off-chip sample heating times and short on-chip heating times, compared with bead-beating.

To determine whether, given enough time, thermal lysis can perform comparably to bead beating, a set of samples was incubated off-chip for extended time periods using a benchtop thermocycler (Figure 7). At very long incubation times (>>10 min), thermal lysis approaches the DNA yield of PCR-available DNA obtained by bead-beating. Significantly, a more typical protocol for benchtop thermal lysis (5 min. incubation at 90 °C using a heating block and individual microcentrifuge tubes) yields only 25–50% of the maximum DNA quantity obtained by bead-beating. This set of measurements makes clear that DNA release by 90 °C treatment at very short timescales cannot be expected to match the performance of bead-beating. 

## 4. Discussion, Conclusions and Outlook

**Figure 8:**
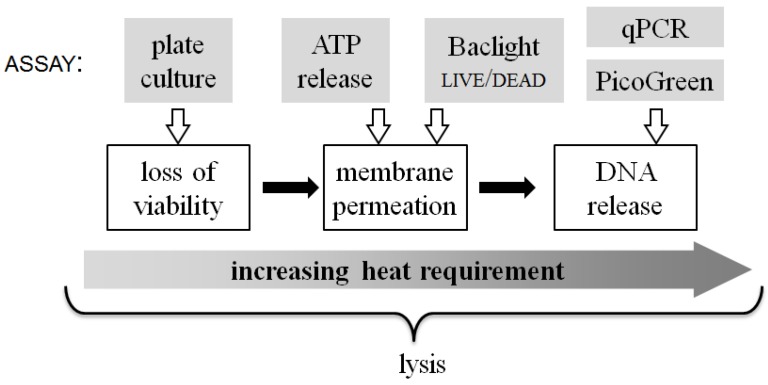
A qualitative scale of biophysical changes that occur in bacterial cells during lysis and the relative energy input required to attain them. At the top of the figure, each assay used in this work is linked to the approximate lysis step that it quantifies.

The multiple assays used in this work to evaluate the results of on-chip thermal lysis hint at the physical changes that take place in *E. coli* due to heat treatment. Figure 8 summarizes the qualitative relationship between the energy input required to achieve different aspects of the lysis process. The three white-boxed cell state changes shown in Figure 8 are, at times, used interchangeably with the term “lysis”, but how the steps relate to each other is seldom considered. For instance, even mild (30 °C) heat-treatment of *E. coli* significantly reduces their viability, as does rapid treatment at high temperature (90 °C). Loss of viability is commonly understood to be closely coupled to membrane compromise (indeed the BacLight live/dead assay assumes that the latter is a proxy for the former). However, significant viability loss takes place at shorter treatment times and at lower temperatures, before substantial quantities of molecules diffuse out of or into the compromised membrane. 

Likewise, membrane permeability itself appears to be more complex than a single-step process. This is indicated by the fact that the release of ATP from the permeabilized cells is complete and dramatic with short treatment at 90 °C and significant even at 70 °C, whereas the entry of propidium iodide (PI) and binding to the DNA requires greater thermal energy to accomplish. The molecular weights (ATP: 551 g/mol, PI: 668 g/mol) and solubilities (ATP: 50 mg/mL, PI: 67 mg/mL) of the two molecules are similar, but it can be speculated that another parameter likely affecting results from these assays is the measurement timescale. The Baclight live/dead assay is observed in near-real-time, as the samples flow through the chip, such that transport and binding processes may not have reached steady state. In contrast, ATP release is quantified minutes after sample collection and centrifugation post-chip, providing ample time for equilibration and completion of reactions. The detailed reasons for the difference in these assay results will need to be investigated in further studies. 

Finally, quantitation of released DNA indicates that liberating the nucleic acid content of the *E. coli* cells requires the greatest energy input. The data suggest that progressively longer heating causes ever more cell disruption and release of DNA. The measurements using PicoGreen dye support this overall trend, though the drop-off in fluorescence for high temperatures and long incubation times still remains to be understood. When measured by qPCR, DNA release due to rapid thermal treatment seems relatively low compared to bead-beating. We believe this is at least partly an artifact due to bead-beating producing on average smaller DNA fragment sizes (maximum size 5–20 kb [25,26]). Since we expect on-chip thermal lysis to generate longer fragment sizes, up to fully-intact genomic DNA, it is not surprising that relatively more of this high-molecular-weight DNA is retained by the spin-filter together with any un-lysed cells, reducing PCR-available DNA post filtration. It is worth noting that rRNA is another common target molecule for diagnostic assays in bacteria. Since rRNA is both more abundant than DNA (>10^3^ copies per cell) and made of shorter segments (1.5 and 2.9 kb) tightly coupled to proteins, its release is likely to be quite different than DNA. This is another aspect of the thermal lysis process that would be useful to explore.

In conclusion, although on-chip thermal lysis for <60 s underperforms 5–10 min tube-based benchtop heating protocols, the integration and automation potential of the on-chip method outweighs these drawbacks in many contexts. For instance, this approach has been successfully integrated with on-chip bacterial capture and an *in situ* hybridization assay for bacterial detection [27]. More broadly, for other rapid diagnostic purposes, the duration and intensity of heating can be matched to provide lysis appropriate for the target molecules, whether nucleic acids or proteins. It is important to note that Gram-positive bacterial cells have a thicker peptidoglycan layer in their wall than do Gram-negative bacteria, making them more difficult to lyse. *Mycobacterium* spp. have even tougher cell walls. We can therefore expect that such cell types will require significantly more heat energy input than *E. coli* to lyse successfully.

This initial study of *E. coli* lysis performance under different assay conditions suggests that the utility of on-chip thermal lysis should be explored further for a variety of scenarios, target cell types and applications. This technology is particularly suitable in situations where rapid membrane permeabilization or sterilization are the primary aims or where integration with other on-chip technologies is paramount. 
